# Proline Concentration and Its Metabolism Are Regulated in a Leaf Age Dependent Manner But Not by Abscisic Acid in Pea Plants Exposed to Cadmium Stress

**DOI:** 10.3390/cells10040946

**Published:** 2021-04-20

**Authors:** Edyta Zdunek-Zastocka, Agnieszka Grabowska, Beata Michniewska, Sławomir Orzechowski

**Affiliations:** Department of Biochemistry and Microbiology, Warsaw University of Life Sciences—SGGW, Nowoursynowska 159, 02-776 Warsaw, Poland; agnieszka_grabowska@sggw.edu.pl (A.G.); beata_michniewska@sggw.edu.pl (B.M.); slawomir_orzechowski@sggw.edu.pl (S.O.)

**Keywords:** abscisic acid, cadmium, heavy metals, malondialdehyde, *Pisum sativum* L., pyrroline-5-carboxylate synthetase, proline dehydrogenase, proline transporters

## Abstract

The accumulation of proline is one of the defense mechanisms of plants against the harmful effects of adverse environmental conditions; however, when pea plants were treated for 12 h with CdCl_2_, the proline concentration decreased in the youngest A (not expanded) and B1 (expanded) leaves, and did not change significantly in the B2 (mature, expanded) or C (the oldest) leaves. After 24 h of cadmium (Cd) stress, the proline concentration remained low in A and B1 leaves, while in B2 and C leaves, it increased, and after 48 h, an increase in the proline concentration in the leaves at each stage of development was observed. The role of proline in the different phases of plant response to the Cd treatment is discussed. Changes in proline accumulation corresponded closely with changes in the transcript levels of *PsP5CS2*, a gene encoding D1-pyrroline-5-carboxylate synthetase involved in proline synthesis, and *PsPDH1*, a gene encoding proline dehydrogenase engaged in proline degradation. CdCl_2_ application induced the expression of *PsProT1* and *PsProT2*, genes encoding proline transporters, especially during the first 12 h of treatment in A and B1 leaves. When the time courses of abscisic acid (ABA) and proline accumulation were compared, it was concluded that an increase in the proline concentration in the leaves of Cd-treated pea plants was more related to a decrease in chlorophyll concentration (leaves B2 and C) and an increase in the malondialdehyde level (A and B1 leaves) than with an increase in ABA concentration alone. Exogenous application of ABA (0.5, 5, 50 µM) significantly increased the proline concentration in the A leaves of pea plants only, and was accompanied by an elevated and repressed expression of *PsP5CS2* and *PsPDH1* in these leaves, respectively. The presented results suggest that under Cd stress, the accumulation of proline in leaves of pea plants may take place independently of the ABA signaling.

## 1. Introduction

Over the past few decades, increased anthropogenic activity, rapid industrialization, and the use of metal-based pesticides have led to an increased contamination of soil and water with heavy metals, which in turn causes toxicity to living organisms [1,2]. Among them, cadmium (Cd) is one of the most common heavy metals in a contaminated environment, which negatively affects plant growth and development, even when available in low concentrations. From the soil, Cd can be easily taken up by plants, leading to severe disturbances in plant physiological and biochemical processes [3,4]. Cd inhibits photosynthesis and transpiration in leaves [4], limits water uptake, and disrupts the uptake and movement of mineral nutrients [5]. At the cellular level, Cd toxicity can be explained by its ability to react with the sulfhydryl groups of proteins, replacement of some essential elements at the active sites of enzymes, and replacement of magnesium in both chlorophyll a and b [4,6]. Although Cd is not a redox-active metal, it can induce oxidative stress, possibly by interfering with an antioxidant defense, by increasing nicotinamide adenine dinucleotide phosphate (NADPH) oxidase activity, or by disrupting the electron transport chain [7,8].

To counteract the deleterious effects of adverse environmental conditions, plants have evolved multiple defense mechanisms. Among these, a crucial role is played by the synthesis of protective compounds, including specific amino acids such as proline, peptides such as glutathione and phytochelatins, and polyamines such as putrescine [9,10]. Stress-induced proline accumulation has been confirmed in many plant species and has been associated with adaptation, particularly to osmotic stress caused by drought or salinity or simulated by polyethylene glycol (PEG) [9,11]. Apart from being one of the most widespread compatible osmolytes, proline may contribute to scavenging reactive oxygen species (ROS), stabilizing proteins and subcellular structures, and regulating intracellular redox potential, and it may function as a signaling molecule or heavy metal chelator [9,11,12,13,14].

Under stress conditions, proline is synthesized mainly from glutamate, which is reduced to glutamate semialdehyde by the rate-limiting D1-pyrroline-5-carboxylate synthetase (P5CS), and then spontaneously converted to pyrroline-5-carboxylate (P5C) and reduced to proline by P5C reductase [12,15]. In plants, P5CS is typically encoded by two genes that exhibit distinctive expression patterns, suggesting different metabolic functions. In *Arabidopsis thaliana*, transcription of *AtP5CS1* is induced under drought, salinity and phosphate deficiency, whereas *AtP5CS2* is upregulated during incompatible plant–pathogen interactions [16,17,18,19,20,21]. Moreover, *p5cs1* mutants have shown a reduction in stress-induced proline accumulation and hypersensitivity to salt stress, while *p5cs2* mutation caused embryo abortion upon seed development [22]. In *Medicago truncatula*, transcript abundance of *MtP5CS1* was unaffected by osmotic stresses, in contrast to *MtP5CS2*, which expression was strongly induced in shoots of salt-stressed plants [23].

Proline catabolism occurs through the action of proline dehydrogenase (PDH), which oxidizes proline to P5C, and by P5C dehydrogenase (P5CDH) converting P5C to glutamate. In *Arabidopsis* and *Medicago sativa*, PDH is encoded by two genes, while *P5CDH* exists as a single copy gene [24,25]. During salt stress, expression of both *MsPDH*s strongly decreased and was closely correlated with proline accumulation, whereas transcript levels of *MsP5CDH* remained unchanged [25]. In *Arabidopsis*, both *ProDH*s were induced by exogenous proline; however, NaCl was found to repress *ProDH1* and induce *ProDH2* [21,24,26,27]. Interestingly, the *Arabidopsis pdh1* mutant displayed hypersensitivity to proline despite the presence of *AtPDH2*, emphasizing the role of *AtPDH1* in proline catabolism [28].

Based on current knowledge, the reactions catalyzed by P5CS and PDH are the rate-limiting steps in proline biosynthesis and degradation, respectively, under stressful conditions [12]. However, the intracellular proline concentration is the cumulative result of not only the rates of biosynthesis and degradation, but also transport between cells and organs [29,30]. The three *Arabidopsis* proline transporters (ProTs) are localized at the plasma membrane and mediate the transport not only of proline, but also of glycine betaine and γ-aminobutyric acid [31]. Transcripts of *AtProT1* have been found in the phloem or phloem parenchyma cells, indicating a role in the long-distance transport of solutes [31], whereas expression of *AtPro2* as well as *GmProT1* and *GmProT2* in alfalfa has been found to be strongly induced in response to salt and drought, implying an important role in nitrogen distribution under stress conditions [31,32].

Although accumulation of proline under osmotic stress is a well-known phenomenon, the results describing the changes in proline concentration due to cadmium toxicity are often contradictory, especially in the leaves. Cadmium has been found to significantly increase proline accumulation in the leaves of sunflower [33], cucumber [34], and bean seedlings [35], while reduced proline concentration was observed in the leaves of oilseed rape [36], wheat [37], and hybrid poplar [38]. The influence of cadmium on proline concentration was investigated after several days or weeks of treatment; hence, differences may have resulted from the differing roles of this multifunctional amino acid [11] at individual stages of adaptation to unfavorable conditions. There is a shortfall of information on the dynamics of changes in proline concentration in the first 48 h of cadmium exposure, and even less is known about the metabolism and transport of proline under these conditions. Moreover, the analysis of leaves at different stages of development would provide new information enabling an understanding of the role of proline in the early response of a plant to Cd ions.

Some results have demonstrated that accumulation of proline under osmotic stress can be mediated by abscisic acid (ABA) [39,40]. Under drought stress, transgenic *Arabidopsis* and petunia plants overexpressing *NCED* (9-cis-epoxycarotenoid dioxygenase), a rate-limiting gene in ABA biosynthesis in response to stress, accumulated significantly more proline than did wild-type (WT) plants [41,42]. Moreover, treatment with norflurazon, an ABA synthesis inhibitor, inhibited proline synthesis and aggravated hypoxia-induced oxidative damage in rice roots [43]. However, the use of ABA-insensitive mutants (*abi1-1*, *abi2-1*, *abi3*, *abi4*, *abi5*) shows that proline accumulation under low water potential was dependent not only on the ABA concentration, but also on the plant sensitivity to this hormone [44]. Furthermore, in ABA-deficient *aba1-1* mutants, the expression of the genes involved in proline biosynthesis was independent of the endogenous level of ABA in *Arabidopsis* under cold and osmotic stresses [45]. It was also reported that ABA alone was not able to duplicate drought-induced proline accumulation [46], and the application of exogenous ABA did not increase the proline concentration in barley or spinach [47]. Therefore, at least under drought, cold, or PEG-induced osmotic stresses, proline accumulation is mediated both by ABA-dependent and ABA-independent signaling pathways. Under conditions of Cd toxicity, however, it is not known whether there are any interactions between ABA and proline accumulation or whether the metabolism of proline and its transport are ABA-independent.

Therefore, the present study investigates the possible mediating role of ABA in the regulation of proline accumulation and its metabolism in the early stages of Cd toxicity in pea leaves at various stages of development. First, the dynamics of changes in ABA concentration with changes in proline accumulation and the expression of genes related to its metabolism and transport during the first 48 h of Cd treatment were investigated, and relationships between these variables were explored. Subsequently, the effects of exogenous ABA application on proline concentration and expression of genes related to its metabolism and transport were examined.

## 2. Materials and Methods

### 2.1. Plant Material and Experimental Conditions

Seeds of *Pisum sativum* L. (cv. Iłówiecki) were germinated in moist vermiculite, and the 12-day-old seedlings were transferred to containers filled with aerated 1/2 Hoagland medium [48]. After two days, the medium was supplemented with CdCl_2_ (50 µM) or ABA (0.5, 5, or 50 µM). Plants grown in a medium without CdCl_2_ or ABA served as controls. After 12, 24, and 48 h of the treatments, plants (twelve plants per treatment) were harvested and separated into roots and leaves (leaflets plus stipule). The leaves were grouped according to the stage of development. The oldest true leaves were collected from the first node from the bottom and were termed C leaves. Leaves taken from the second and third nodes were termed B2 leaves. The youngest fully expanded leaves were collected from the fourth node and were termed B1 leaves, while the youngest unexpanded leaves were taken from the fifth node and were termed A leaves. The root and leaf samples were frozen in liquid N_2_ immediately after harvest and stored at –80 °C until use.

The experiments were conducted in a plant growth chamber (the MLR-352H Climate Chamber, PHC Biomedical—formerly Panasonic Biomedical) at a humidity of approximately 60% and light intensity of 300 µmol s^−1^ m^−2^. The air temperature was 22 °C (16 h) during the day and 19 °C (8 h) at night. The experiment (all treatments) was repeated independently at least three times, giving at least three independent biological replicates. A minimum of two technical replicates were performed for each biological replicate.

### 2.2. Total RNA Extraction and cDNA Synthesis

Total RNA was isolated according to Chomczynski and Sacchi [49], except that RNA was precipitated for 2 h on ice by the addition of 0.3 volume of 10 M LiCl. The isolated RNA was treated with DNase I (Thermo Scientific) and a total of 2 µg of RNA was reverse transcribed in a final volume of 10 μL using the Transcriptor First Strand cDNA Synthesis Kit (Roche) according to the manufacturer’s protocol.

### 2.3. Identification of Nucleotide Sequences of Genes Involved in Proline Metabolism and Transport

Internal fragments of the analyzed *Pisum sativum* genes (*PsP5CS1*, *PsP5CS2, PsPDH1*, *PsProT1*, *PsProT2*) were amplified using Q5 High-Fidelity DNA Polymerase (New England BioLabs) and primers designed from known sequences of *Medicago truncatula P5CS1* (AJ278818), *P5CS2* (JN809240), *PDH1* (XM_013595747), *ProT1* (XM_003600790), and *ProT2* (XM_013602264). Primers are listed in Supplementary Table S1. PCR conditions were as follows: 30 s at 98 °C; 40 cycles of 10 s at 98 °C, 30 s at 56 °C (*PsPDH1*) or 58 °C (*PsP5CS1, PsP5CS2, PsProT1, PsProT2*), and 1 min at 72 °C; followed by a final extension for 10 min at 72 °C. PCR products were cloned into the pJET1.2/blunt cloning vector (Thermo Scientific), amplified in *E. coli* JM109, and then sequenced at the DNA Sequencing and Oligonucleotide Synthesis Laboratory at The Institute of Biochemistry and Biophysics, Polish Academy of Sciences. At least six clones of each PCR product were sequenced and analyzed.

### 2.4. Gene Expression Analysis

Real-time PCR was performed on a LightCycler^®^ 96 instrument (Roche Diagnostics). The reaction mixture contained 5 μL of SYBR Green I Master (Roche Diagnostics), 3 μL of cDNA template (equivalent to 10 ng of total RNA), and 0.5 μM of each primer in a total volume of 10 μL. Thermal cycling conditions were as follows: 10 min at 95 °C, 40 cycles of 15 s at 95 °C, 15 s at 63 °C, and 20 s at 72 °C. Melting curves (95 °C for 10 s, 65 °C for 1 min, and 97 °C for 1 s in continuous acquisition mode) were generated for each reaction to ensure amplification specificity. All reactions were performed in triplicate. The relative expression levels of target genes were calculated using the 2^−ΔΔCt^ method [50]. The genes encoding β-tubulin and actin were used as internal controls [51]. The absence of genomic DNA in RNA samples was verified using primers designed to different exons of gene encoding GAPDH (glyceraldehyde-3-phosphate dehydrogenase) [51]. All primers used for real-time PCR are listed in Supplementary Table S2. All reactions were performed at least in triplicate for each of the three biological repetitions.

### 2.5. Determination of ABA and Proline Concentrations

Abscisic acid was extracted from pea tissues as described by Zdunek-Zastocka and Grabowska [52]. Briefly, 200 mg of frozen leaves were ground to a fine powder with liquid nitrogen and mixed with 1.4 mL of a solution consisting of 80% methanol, 2% glacial acetic acid, and butylated hydroxytoluene (20 mg L^−1^). The extracts were shaken at 4 °C in the dark for 24 h and then centrifuged at 12,000× *g* and 4 °C for 20 min. Supernatants were diluted 25 times in Tris-buffered saline, TBS (150 mM NaCl, 25 mM Tris-HCl, pH 7.5), and 100 µL of the diluted extracts were used for the quantification of ABA using the Phytodetek ABA enzyme immunoassay test kit (Agida) according to manufacturer’s instructions.

Extraction and measurement of free proline in pea tissues were performed according to the acid ninhydrin method described by Bates et al. [53]. Briefly, samples of 100 mg frozen leaves were homogenized with 2 mL of 3% sulphosalicylic acid in a pre-chilled mortar and pestle. The extracts were shaken for 30 min at 750 rpm. The residues were removed by centrifugation at 12,000× *g* for 20 min. Supernatants (0.8 mL) were mixed with an equal volume of an acid-ninhydrin reagent (1.25 g ninhydrin, 30 mL of glacial acetic acid, and 20 mL of 2 M orthophosphoric acid) and incubated for 60 min in boiling water. After cooling, the reaction mixture was extracted with 2 mL of toluene, mixed vigorously and left at room temperature for 20 min until separation of the two phases occurred. The absorbance of toluene phase was measured at 520 nm using pure toluene as a blank.

### 2.6. Determination of Chlorophyll and MDA Concentrations

Total chlorophyll was extracted using the dimethyl sulfoxide (DMSO) method described by Hiscox and Israelstam [54]. Frozen pea leaves (50 mg) were mixed with 2 mL of dimethyl sulfoxide (DMSO) and incubated for 60 min at 65 °C. The residues were removed by centrifugation at 12,000× *g* for 10 min, and the absorbance of the chlorophyll extracts was measured at 649 and 665 nm against DMSO as a blank.

The amount of malondialdehyde (MDA) was measured according to the thiobarbituric acid test [55]. Samples of 150 mg frozen leaves were homogenized with 1.5 mL 0.1 M potassium phosphate buffer, pH 6.8 in a pre-cooled mortar and pestle. The homogenates were centrifuged at 10,000× *g* for 15 min. Supernatants were mixed in 1:1 proportions with TBA (0.5% thiobarbituric acid in 15% trichloroacetic acid) and incubated for 20 min at 100 °C. After cooling, the solution was centrifuged at 10,000× *g* for 10 min and the absorbance of the supernatant was measured at 532 nm and 600 nm.

### 2.7. Statistical Analysis

The statistical analysis was performed using the Statistica 9.1 program. The conformity of data with normal distribution was verified using the Shapiro-Wilk test, and the equality of variances between the two data sets was verified using Levene’s test. Thereafter, the parametric Tukey’s test (almost all analyses) or the non-parametric Kruskal–Wallis test (analyses of the *PsP5CS1* expression under Cd treatment) was used, accordingly.

## 3. Results

### 3.1. Identification of Nucleotide Sequences of Genes Involved in the Metabolism and Transport of Proline

A nucleotide sequence fragment (711 bp), sharing a high percentage identity with the sequences of *P5CS1* genes from *Medicago truncatula* (*MtP5CS1*, AJ278818, 92%) [23,56], *Medicago sativa* (*MsP5CS1*, X98421, 92%) [57], *Brassica napus* (*BnP5CS1*, AF314811, 75%), and *Arabidopsis thaliana* (*AtP5CS1*, NM_129539, 73%) [16,22], was identified in pea plants and designated *Pisum sativum P5CS1* (*PsP5CS1*, MW030636) (Supplementary Figure S1).

A 945-bp nucleotide sequence fragment, sharing a high percentage identity with the sequences of *P5CS2* genes from *Medicago truncatula* (*MtP5CS2*, JN809240, 83%) [23,56], *Arabidopsis thaliana* (*AtP5CS2*; NM_115419, 72%) [16,22], and *Brassica napus* (*BsP5CS2*, AF314812, 67%), was identified in pea plants and designated *Pisum sativum P5CS2* (*PsP5CS2*, MW423825) (Supplementary Figure S2).

A nucleotide sequence fragment (471 bp) that shared a high percentage identity with the sequences of stress-downregulated *PDH* genes from *Medicago truncatula* (*MtPDH1*, XM_013595747, 87%) [56], *Medicago sativa* (*MsPDH1*, AY556386, 80%; *MsPDH2*, AY615900, 77%) [25], *Nicotiana tabacum* (*NtPDH2*, AY639146, 69%) [58], and *Arabidposis thaliana* (*AtPDH1*, NM_001339059, 66%) [59] was identified in pea plants and designated *Pisum sativum PDH1* (*PsPDH1*, MW183670) (Supplementary Figure S3).

A 617-bp nucleotide sequence fragment that shared 84% identity with the *MtProT1* (XM_003600790) and *GmProT1* (XM_003552545) genes from *Medicago truncatula* and *Glycine max*, respectively, and 70% identity with the *AtProT* genes from *Arabidopsis thaliana* (*AtProT1*, X95737; *AtProT2*, X95738; *AtProT3*, NM_129215) was identified in pea plants and designated *Pisum sativum ProT1* (*PsProT1*, MW030634) (Supplementary Figure S4). The sequence shared less than 60% identity with the sequences of *MtProT2* (XM_013602264) and *GmProT2* (XM_014775460). Additionally, a 460-bp nucleotide sequence fragment that shared 88% identity with the sequence of *MtProT2* and 81% identity with the sequence of *GmProT2* was identified in pea plants and designated *Pisum sativum ProT2* (*PsProT2*, MW030635) (Supplementary Figure S5). The sequence shared less than 60% identity with the sequences of *MtProT1*, *GmProT1*, and all three *AtProT*s.

### 3.2. MDA and Chlorophyll Concentration as Affected by Cadmium Stress

Levels of chlorophyll and malondialdehyde (MDA), an end product of lipid peroxidation [55], can reflect the degree of stress/membrane damage induced by Cd. Therefore, changes in the chlorophyll (a + b) and MDA concentrations over time were analyzed in the leaves of pea plants after 12, 24, and 48 h of growth on media containing 50 µM CdCl_2_. The analyses were performed on leaves at different stages of development: the youngest unexpanded leaves (A), the youngest fully expanded leaves (B1), the fully expanded mature leaves (B2), and the oldest true leaves (C).

**Figure 1:**
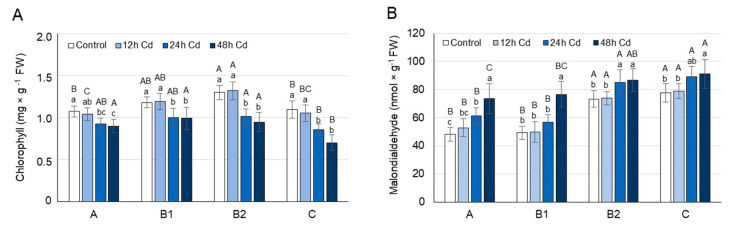
Changes in the chlorophyll (**A**) and malondialdehyde (**B**) concentration in the leaves of pea plants exposed to cadmium (Cd) treatment. Cadmium was applied as 50 µM CdCl_2_. A, the youngest unexpanded leaves; B1, the youngest fully expanded leaves; B2, fully expanded mature leaves; and (C), the oldest true leaves. The results are the means (±SD) of three biological replicates. Significant differences (at least *p* < 0.05) between the means are shown above the columns by different letters: lowercase between different time points in a group of leaves with the same stage of development, and capital between leaves with different stages of development but at the same time point.

Chlorophyll concentration did not change significantly in the leaves of Cd-treated plants after 12 h (Figure 1A); however, after 24 h, it decreased by 15% in A and B1 leaves, and by 22% in B2 and C leaves. After 48 h of Cd treatment, the concentration of chlorophyll in A and B1 leaves decreased as before, by approximately 15%, while in leaves B2 and C, it declined by 27% and 36%, respectively.

Under control conditions, the MDA concentration was approximately 55% higher in B2 and C leaves than in A and B1 leaves. The MDA concentration did not change significantly in the leaves of pea plants after 12 h of Cd stress (Figure 1B); however, after 24 h of growth on CdCl_2_, it increased by about 25% in A leaves and by 15% in B and C leaves. After 48 h of Cd treatment, the MDA concentration increased in A and B1 leaves by approximately 55%, and in B2 and C leaves, as before, by approximately 15%.

### 3.3. Proline Concentration and its Metabolism as Affected by Cadmium Stress

Proline concentration and the expression of genes involved in its metabolism (*PsP5CS1*, *PsP5CS2*, *PsPDH1*) and transport (*PsProT2, PsProT2*) were studied in the leaves of pea plants after 12, 24, and 48 h of growth on media containing 50 µM CdCl_2_. The analyses were performed on leaves at various stages of development.

**Figure 2:**
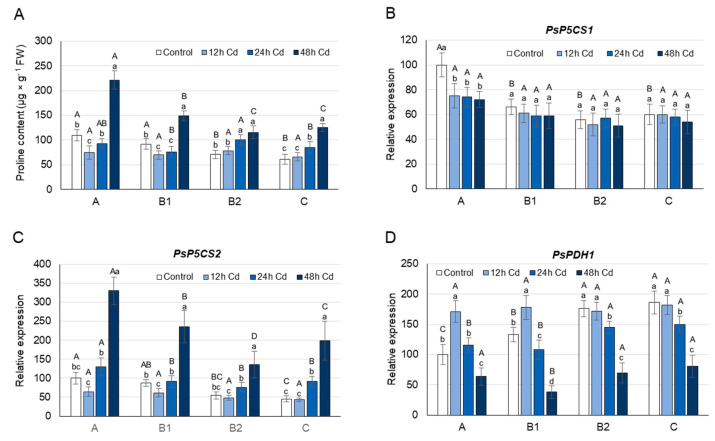
Changes in the proline concentration (**A**) and transcript level of *PsP5CS1* (**B**), *PsP5CS2* (**C**), and *PsPDH1* (**D**) after 12, 24, and 48 h of Cd treatment. Cadmium was applied as 50 µM CdCl_2_. The relative mRNA level in individual leaves was expressed in relation to that in A leaves of control plants, set to 100, after normalization to reference genes. A, the youngest unexpanded leaves; B1, the youngest fully expanded leaves; B2, fully expanded mature leaves; and C, the oldest true leaves. The results are the means (±SD) of three biological replicates. Significant differences (at least *p* < 0.05) between the means are shown above the columns by different letters: lowercase between different time points in a group of leaves with the same stage of development, and capital between leaves with different stages of development but at the same time point.

**Figure 3:**
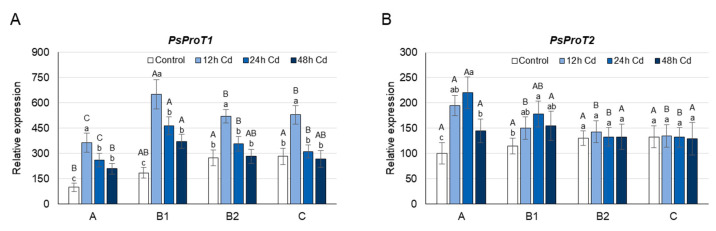
Changes in the transcript level of *PsProT1* (**A**) and *PsProT2* (**B**) after 12, 24, and 48 h of Cd treatment. Cadmium was applied as 50 µM CdCl_2_. The relative mRNA level in individual leaves was expressed in relation to that in A leaves of control plants, set to 100, after normalization to reference genes. A, the youngest unexpanded leaves; B1, the youngest fully expanded leaves; B2, fully expanded mature leaves; and C, the oldest true leaves. The results are the means (±SD) of three biological replicates. Significant differences (at least *p* < 0.05) between the means are shown above the columns by different letters: lowercase between different time points in a group of leaves with the same stage of development, and capital between leaves with different stages of development but at the same time point.

Under control conditions, the highest concentration of proline was found in the youngest undeveloped A leaves, while B1, B2, and C leaves contained approximately 15%, 35%, and 45% less proline, respectively (Figure 2A). The highest level of proline in A leaves coincided with the highest transcript level of both *PsP5CS*s (Figure 2B,C). In contrast to the *PsP5CS* expression, the mRNA level of *PsPDH1* in A leaves was the lowest among leaves at different developmental stages (Figure 2D). A leaf age-specific expression profile similar to that of *PsPDH1* was observed for the *PsProT1* and *PsProT2* genes. Thus, transport of proline from B and C leaves could contribute to the highest proline concentration in the youngest A leaves under control conditions (Figure 3A,B).

After 12 h of Cd stress, the proline concentration decreased by approximately 30% and 25% in A and B1 leaves, respectively, while in B2 and C leaves, the proline concentration did not change significantly (Figure 2A). Changes in the proline concentration observed in A and B1 leaves coincided with a 35–30% decrease in the transcript level of *PsP5CS2* in these organs (Figure 2C). In A leaves, additionally, a 25% decrease of *PsP5CS1* transcript level was observed (Figure 2B). The decline in proline concentration observed in A and B1 leaves of Cd-treated plants may also be due to the higher activity of PsPDH1, whose transcript level increased under Cd treatment by 70% and 35%, respectively (Figure 2D). Cadmium treatment also caused changes in the expression of both *PsProT*s, especially in the young A and B1 leaves, which would contribute to the enhanced translocation of proline from or to these organs (Figure 3A,B). The *PsProT1* transcript level increased by approximately 300% in A leaves, by 150% in B1 leaves, and by 75% in B2 and C leaves, while the *PsProT2* mRNA level rose by 100% and 30% in A and B1 leaves, respectively, and did not change significantly in B2 and C leaves (Figure 3A,B).

After 24 h of growth on the CdCl_2_ medium, the decrease in proline concentration was observed again in A and B1 leaves; however, the decline was smaller than after 12 h (Figure 2A). On the other hand, the proline concentration in B2 and C leaves was approximately 35% higher in Cd-treated plants than in plants grown under control conditions, and was accompanied by 30–40% higher *PsP5CS2* transcript levels in these organs (Figure 2C). The expression of *PsP5CS1* was not influenced by Cd ions in B1, B2, or C leaves, while in A leaves, it decreased as after 12 h of Cd treatment by 25% (Figure 2B). The increase in proline concentration observed in B2 and C leaves after 24 h of Cd treatment coincided with a 20% lower level of *PsPDH1* mRNA in these organs (Figure 2D). The proline concentration in B2 and C leaves of Cd-treated plants could also be enhanced by the transport of proline from A and B1 leaves, where the expression of *PsProT1* and *PsProT2* remained higher by 150% in the case of *ProT1*, and by 50–120% in the case of *ProT2*. In turn, the level of *PsProT1* mRNA in B2 and C leaves was significantly less affected than after 12 h of Cd treatment, and the level of *PsProT2* mRNA, similarly to 12 h of Cd stress, did not change significantly (Figure 3A,B).

After 48 h of Cd stress, an increase in proline concentration was observed in leaves at each stage of development (Figure 2A). In A and C leaves, proline concentration in Cd-treated plants was higher than in controls by approximately 100%, and in B1 and B2 leaves, it was higher than in controls by approximately 65%. Higher proline concentration was accompanied by a higher expression of *PsP5CS2* and a lower transcript level of *PsPDH1* (Figure 2C,D). The mRNA level of *PsP5CS2* increased by more than 200% in A and C leaves, and by approximately 160% in B2 and C leaves, while the transcript level of *PsPDH1* decreased by 60–80% in leaves of each developmental stage. After 48 h of Cd stress, the expression of genes encoding proline transporters did not change significantly in B2 or C leaves, while in A and B1 leaves, it was still significantly higher (by 100% in the case of *PsProT1* and by 40% in the case of *PsProT2*) than under control conditions (Figure 3A,B).

### 3.4. ABA Concentration as Affected by Cadmium Stress

**Figure 4:**
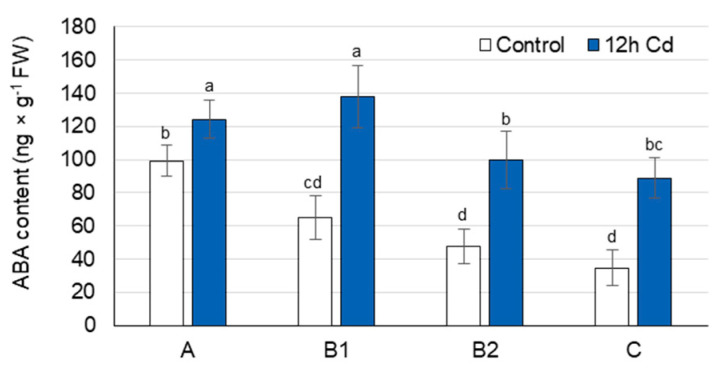
Changes in the abscisic acid concentration after 12 h of Cd treatment. Cadmium was applied as 50 µM CdCl_2._ A, the youngest unexpanded leaves; B1, the youngest fully expanded leaves; B2, fully expanded mature leaves; and C, the oldest true leaves. The results are the means (±SD) of three biological replicates. Different letters above the columns indicate significant differences between the means (*p* < 0.05).

Under control conditions, the ABA concentration was highest in the youngest A leaves, lower in B1 and B2 leaves, while the oldest C leaves contained only 40% of the ABA found in A leaves (Figure 4). Under Cd stress, the ABA concentration in pea leaves increased significantly after only 12 h of treatment, and a similar trend of changes persisted after 48 h (Figure 4 and Figure S6). The greatest increase in ABA concentration (2.6-fold) was observed in the oldest C leaves, although an approximately 2-fold increase was also found in B1 and B2 leaves. In A leaves, the ABA concentration only increased by approximately 20% as a result of Cd treatment.

### 3.5. Proline Concentration and Its Metabolism as Affected by Exogenous ABA

Since the changes in ABA accumulation observed in pea leaves under Cd stress did not correspond clearly with the observed changes in proline concentration, we investigated the effect of exogenously applied ABA on proline concentration and the expression of genes involved in its metabolism and transport.

**Figure 5:**
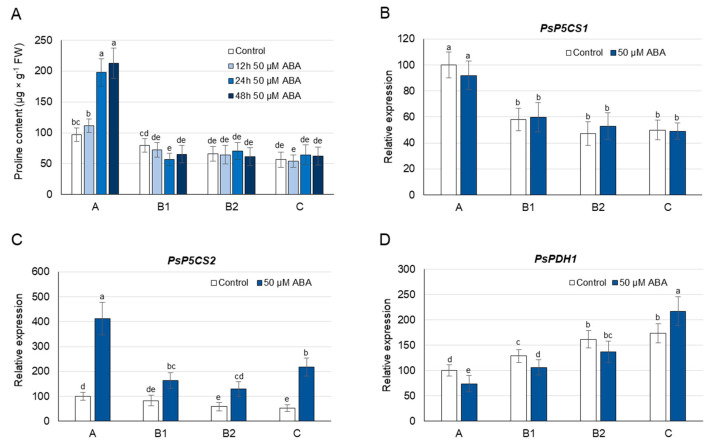
Changes in the proline concentration (**A**), abscisic acid (ABA) concentration (**B**), and transcript levels of *PsP5CS2* (**C**) and *PsPDH1* (**D**) after 24 h of ABA treatment. ABA was applied at a concentration of 50 µM. The relative mRNA level in individual leaves was expressed in relation to that in A leaves of control plants, set to 100, after normalization to reference genes. A, the youngest unexpanded leaves; B1, the youngest fully expanded leaves; B2, fully expanded mature leaves; and C, the oldest true leaves. The results are the means (±SD) of three biological replicates. Different letters above the columns indicate significant differences between the means (*p* < 0.05).

**Figure 6:**
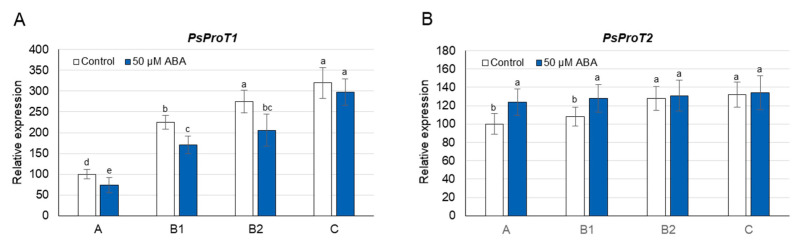
Changes in the transcript levels of *PsProT1* (**A**) and *PsProT2* (**B**) after 24 h of ABA treatment. ABA was applied at a concentration of 50 µM. The relative mRNA level in individual leaves was expressed in relation to that in A leaves of control plants, set to 100, after normalization to reference genes. A, the youngest unexpanded leaves; B1, the youngest fully expanded leaves; B2, fully expanded mature leaves; and C, the oldest true leaves. The results are the means (±SD) of three biological replicates. Different letters above the columns indicate significant differences between the means (*p* < 0.05).

During growth on 50 µM ABA, a significant increase in proline concentration was observed after only 24 h; however, this occurred exclusively in A leaves (2-fold) and also persisted after 48 h (Figure 5A) and 72 h (Supplementary Figure S7). When pea plants were grown on media containing lower concentrations of ABA (0.5, 5 µM), an increase in proline concentration was observed again only in A leaves, and it was less significant than during growth on the 50 µM ABA (Supplementary Figure S8). Thus, the 50 µM concentration of ABA was selected for the gene expression analyses. After 24 h of 50 µM ABA treatment, the concentration of this hormone increased 2–3 times in A and B leaves, and over 7 times in C leaves (Supplementary Figure S9); however, the transcript level of *PsP5CS1* did not change significantly in any of these organs (Figure 5B). In turn, the expression of *PsP5CS2* increased not only in A leaves (4-fold), where ABA-induced proline accumulation occurred, but also in B (2-fold) and C (4-fold) leaves (Figure 5C). The 50 µM ABA treatment decreased the expression of *PsPDH1 (*except for C leaves) and of *PsProT1* in leaves of each developmental stage (Figure 5D and Figure 6A), and increased the transcript level of *PsProT2* in A and B1 leaves (Figure 6B). The above analyses show that ABA induces proline accumulation only in the youngest A leaves; however, its synthesis involving *PsP5CS2* can occur in leaves of each developmental stage. At the same time, ABA treatment reduces both proline degradation (except for C leaves) and proline translocation mediated by *PsProT1*; however, the transport of proline from or to A and B1 leaves by *PsProT2* was increased.

## 4. Discussion

The accumulation of free proline varies considerably in different plant organs, depending both on the developmental stage of the plant and on the environmental stimuli [12,13]. Proline concentration is usually higher in reproductive organs than in vegetative tissues, and increases significantly in actively dividing cells [12,29,60]. When leaves of control pea plants were analyzed according to their stage of development, the highest concentration of proline was found in the youngest undeveloped A leaves, and it gradually decreased through B leaves towards old C leaves (Figure 2A). Similar results were obtained for *Brassica napus*, where four leaf ranks with gradually increasing source status and gradually decreasing proline concentration were identified from the top to the bottom of the plant axis [61]. In accordance with the observed decrease in proline concentration, the expression of four *BnaP5CS1* genes was significantly lower in senescent leaves, which have a strong source leaf status, compared with young growing leaves. In turn, the expression of all *BnaP5CS2*s did not differ significantly between the individual leaf ranks. In pea plants, the expression of both *PsP5CS1* and *PsP5CS2* was the highest in the youngest A leaves and decreases towards old C leaves (Figure 2B,C), suggesting the possible contribution of both genes in the high proline concentration of A leaves. The low proline concentration found in the C leaves of control plants may also be a result of the transport of proline from these leaves, as well as a higher *PsPDH1* expression (Figure 2D and Figure 3). However, when ^15^N-labeling experiments were performed using [^15^N]proline and the leaves of *Brassica napus*, the maximal proline degradation capacity was not different between leaves with very low and very strong source status [61].

Increased proline accumulation is one of the defense mechanisms that plants have developed to counteract the harmful effects of adverse environmental condition stresses such as drought, salinity, and hypoxia [9,11,62,63]. Under Cd stress, however, the changes in proline concentration are often contradictory, especially in the leaves [33,34,35,36,37,64]. Therefore, we examined the changes over time of proline concentration in individual leaves, differing in developmental stage and age, during the first 48 h after the application of Cd ions.

After 12 h of Cd treatment, when the symptoms of damage to cellular structures were not yet observed (Figure 1), a significant decrease in the proline concentration in young A and B1 leaves was noticed (Figure 2A). The decrease in proline concentration was accompanied by a decrease in the transcript level of *PsP5CS2*, a gene involved in its synthesis, as well as an increased expression of *PsPDH1* and *PsProT*s, genes engaged in its degradation and transport, respectively (Figure 2 and Figure 3). The expression of *PsP5CS1* was not influenced by Cd ions in B and C leaves, while in A leaves, it was slightly decreased throughout the entire experimental period (Figure 2B), excluding its key role in the regulation of proline content in response to Cd. In *Arabidopsis* and *Medicago sativa*, PDH is encoded by two genes [24,25]; however, despite many attempts, we were not able to identify the second sequence of the *PsPDH* gene in pea plants. Therefore, it cannot be ruled out that, in addition to *PsPDH1*, another *PDH* gene may be involved in the regulation of proline in response to stress factors. Cd is known to disrupt the uptake and movement of mineral nutrients within plants; it predominantly reduces nitrate uptake and mineral transport from root to shoot, as well as inhibits the activity of the enzymes involved in N assimilation [5,65]. A decrease in NO_3_^−^ uptake and nitrate reductase activity was observed in pea plants as early as 24 h after Cd supply and was more severe at 50 µM than at 10 µM CdSO_4_ [66]. Therefore, in an early plant response to 50 µM Cd, a decrease in free proline concentration, which was observed in A and B1 leaves of pea plants (Figure 2A), may result from low nitrogen availability [67]. It can also be triggered by a greater demand for glutamic acid, the product of proline degradation serving as a N source not only for the synthesis of other amino acids, which are especially needed in young developing leaves, but also for the synthesis of glutathione, certain cofactors, and nucleosides [68]. Moreover, an intensified proline catabolism starting with its oxidation by mitochondrial PDH may provide the energy, in the form of FADH_2_ and NAD(P)H [69,70], necessary to cope with the negative effects of Cd toxicity.

After 24 h of Cd stress, chlorophyll concentration began to decrease in leaves of all developmental stages, with the greatest declines in B2 and C leaves, while MDA levels began to increase, with the largest rise being observed in A leaves (Figure 1). At that time, the proline concentration was still lower in the A and B1 leaves of Cd-treated plants than in controls; however, in B2 and C leaves, it started to increase (Figure 2A). The changes in proline concentration in B2 and C leaves coincided with an elevated expression of *PsP5CS2* and a decrease in the *PsPDH1* transcript level (Figure 2C,D). After 24 h of Cd stress, proline translocation still seems to be important; however, mainly to or from young A and B1 leaves, as a significant increase in the expression of genes related to proline transport was noticeable only in these leaves (Figure 3). It seems that in young A and B1 leaves of Cd-treated plants, proline is still used as a source of nitrogen and energy, while in B2 and C leaves, where the greatest decrease in chlorophyll concentration is noticeable, this amino acid may be necessary to cope with the harmful effects of oxidative stress, which gradually increase after Cd exposure. Proline has been shown to play a protective role in metal-induced lipid peroxidation and to prevent membrane damage [14].

After 48 h of Cd stress, when the rate of lipid peroxidation was very high in A and B1 leaves, the proline concentration in these leaves was also higher in Cd-treated plants than in controls (Figure 1 and Figure 2A), which was accompanied by a large increase in the *P5CS2* transcript level and a reduction in *PsPDH1* expression (Figure 2B,C). Hence, the emerging physiological symptoms of oxidative stress induced by Cd in A and B1 leaves could lead to an equilibrium shift in proline metabolism from the catabolic to the anabolic pathway.

Under drought, salinity, cold, and PEG-induced osmotic stresses, proline accumulation is mediated by both ABA-dependent and ABA-independent signaling pathways [44,45,46,47]. Elevated endogenous ABA concentration after Cd treatment has been detected in *Solanum tuberosum* [71], *Brassica napus* [72], *Malus hupehensis* [73], *Typha latifolia*, and *Phragmites australis* [74] plants; however, the relationship between ABA and proline accumulation under Cd stress is not well understood. Therefore, to investigate a possible mediating role of ABA in the regulation of proline concentration under Cd stress, the time courses of ABA and proline accumulation were first compiled. After 12 h of Cd treatment, the ABA concentration increased by 20% in A leaves and more than doubled in B and C leaves (Figure 4); however, an increase in proline accumulation was not observed in any of these leaves (Figure 2A). In individual leaves, a similar trend of Cd-induced changes of ABA concentration persisted up to 48 h (Supplementary Figure S6). The proline concentration, however, varied depending on the stage of leaf development, and its increase was first observed after 24 h in B2 and C leaves, and only after 48 h in A and B1 leaves. Therefore, an increase in proline concentration in the leaves of Cd-treated pea plants was more highly associated with a decrease in chlorophyll concentration (B2 and C leaves) and an increase in MDA concentration (A and B1 leaves) than with the elevated level of ABA alone, at least in the first 48 h of Cd treatment. Thus, a direct mediating role of ABA in Cd-induced proline accumulation in leaves of pea plants during the early response to Cd treatment could be largely excluded. A positive correlation between ABA concentration and proline accumulation was also not observed in leaves of Cd-treated rice plants. After treatment with Cd, the ABA concentration increased in the leaves of the Cd-tolerant rice cultivar, which did not lead to proline accumulation; on the contrary, in the leaves of the Cd-sensitive cultivar, the proline level increased, but not that of ABA [75].

As discussed above, a positive relationship was not always observed between the ABA concentration and the proline accumulation during the early response to Cd stress in leaves of pea plants; therefore, the effects of exogenously applied ABA on the proline concentration and expression of genes involved in its metabolism and transport were investigated. After 12, 24, 48 and 72 h of phytohormone application (0.5, 5, 50 µM), the proline concentration only increased in A leaves, accompanied by an elevated expression of *PsP5CS2* not only in these organs, but also in B and C leaves (Figure 5A,C, Figures S7 and S8). The transcript level of *PsP5CS1* was not affected by Cd application in any of the pea leaves (Figure 5B). Increases in proline concentration and the *P5CS* transcript level have previously been observed following ABA application in roots of potato (*StP5CS1*) [71], roots and leaves of *Arabidopsis* (*AtP5CS1*) [16,17], and leaves of wheat (*P5CS1*) [76] and rice (*OsP5CS1*) [77]. On the other hand, no increase in proline concentration was reported in spinach or pearl millet [47], and wild-type levels of *P5CS1* transcripts were found in the ABA-deficient mutant *aba2-1*, suggesting the ABA-independent regulation of proline synthesis [45]. Thus, the analysis of leaves at different stages of development would provide additional information enabling an understanding of the role of ABA in the regulation of proline synthesis under Cd stress. Real-time PCR analyses showed that the transcript level of *PsPDH1* was reduced in response to applied ABA in most of the pea leaves (Figure 5D). Similar changes in *PDH* expression have also been noted in the shoots and roots of *Arabidopsis* [17] and in seedlings of *Brassica napus* [78]; however, when rice seedlings were exposed to ABA, the transcript levels of *PDH* did not change. Moreover, it has also been shown that the *PDH* promoter in *Arabidopsis* is induced by exogenously applied Pro but is not affected by ABA [79]. In this way, the *PDH* response to ABA signals still needs to be thoroughly investigated.

In conclusion, the accumulation of proline during the early response to CdCl_2_ in leaves of pea plants may occur independently of ABA signaling. The changes in proline concentration observed after Cd treatment, however, depend on the age of the leaves as well the duration of the stress factor. The concentration of proline in pea leaves is a result of an equilibrium between its synthesis, mediated by *PsP5CS2*, the catabolism, mediated by *PsPDH1*, and transport, mediated by *PsProT1* and *PsProT2*. The expression of *PsP5CS1*, another potential gene involved in proline synthesis, did not correspond to the proline changes during the first 48 h of Cd treatment.

## Data Availability

The data presented in this study are available on request from the corresponding author.

## Supplementary Materials

The following are available online at https://www.mdpi.com/article/10.3390/cells10040946/s1: Table S1: Oligonucleotide sequences of *Medicago truncatula* used as primers for amplification of internal fragments of the *Pisum sativum* genes, Table S2: Primer sequences used for real-time PCR analysis, Figure S1: The alignment of the nucleotide sequence fragment of *Pisum sativum PsP5CS1* (MW030636) with the sequences of *P5CS1* genes from other plants, Figure S2: The alignment of the nucleotide sequence fragment of *Pisum sativum PsP5CS2* (MW423825) with the sequences of *P5CS2* genes from other plants, Figure S3: The alignment of the nucleotide sequence fragment of *PsPDH1* (MW183670) with the sequences of stress-downregulated *PDH* genes from other plants, Figure S4: The alignment of the nucleotide sequence fragment of *PsProT2* (MW030635) with the sequences of *ProT* genes from other plants, Figure S5: The alignment of the nucleotide sequence fragment of *PsProT2* (MW030635) with the sequences *ProT* genes from other plants, Figure S6: Changes in the abscisic acid (ABA) concentration after 48 h of Cd treatment, Figure S7: Changes in the proline concentration after 72 h of abscisic acid (ABA) treatment, Figure S8: Changes in the proline concentration after 24 h of abscisic acid (ABA) treatment, Figure S9: Changes in the abscisic acid concentration after 24 h of abscisic acid (ABA) treatment.
