# Supplementary material for: Proline Concentration and Its Metabolism Are Regulated in a Leaf Age Dependent Manner But Not by Abscisic Acid in Pea Plants Exposed to Cadmium Stress
Source: Cells. 2021 Apr 20;10(4):946. doi: 10.3390/cells10040946 (PMC8073832; doi:10.3390/cells10040946)
Supplement: Supplementary file 1 [file cells-10-00946-s001.zip › cells-1146944-supplementary.pdf]

Proline concentration and its metabolism are regulated in a leaf age dependent manner but not by abscisic acid in pea plants exposed to cadmium stress

Edyta Zdunek-Zastocka*, Agnieszka Grabowska, Beata Michniewska, Sławomir Orzechowski

Department of Biochemistry and Microbiology, Warsaw University of Life Sciences – SGGW, Nowoursynowska 159, 02-776 Warsaw, Poland

*The corresponding author: e-mail address: edyta_zdunek_zastocka@sggw.pl, tel.: +48 22 593 2577; fax: +48 22593 2562.

Table S1. Oligonucleotide sequences of *Medicago truncatula* used as primers for amplification of internal fragments of the *Pisum sativum* genes. F, forward primer; R, reverse primer.

***Pisum sativum* gene**	**Accession number of the *Medicago truncatula* sequence in GenBank database**	**Oligonucleotide sequence (5'->3')**
PsP5CS1	AJ278818	F: CGCCCTGATGCATTGGTACAGATAGC<br>R: CATTGTGAAAAACAGCAGCACTATC
PsP5CS2	JN809240	F: ATGACTGCTAAGGTTAATGCTGCTG<br>R: GATCTTTGTGAACAAGAAGGGTTTCC
PsPDH1	XM_013595747	F: CATGCCAATATTGGTTGATGCTGAACACAC<br>R: GTGCATCTGACATTCCACATAGTTG
PsProT1	XM_003600790	F: GACACATTAGATACAGAGATCTTGCTG<br>R: GCAATGACTGATTGAAGGAAGGCTG
PsProT2	XM_013602264	F: CTATTGTTGGATATTGGGCTTATGG<br>R: CGAAGAGCTGAAATTGTGGTTGC

Table S2. Primer sequences used for real-time PCR analysis. F, forward oligonucleotide; R, reverse oligonucleotide.

**Gene**	**Accesion number of the *Pisum sativium* sequence in GenBank database**	**Oligonucleotide sequence (5'->3')**
PsP5CS1	MW030636	F: CGAAGTGGGAATGGGCTTCTCTTG<br>R: CAACTTAAGTAGCTCGGGTATTTCTGC
PsP5CS2	MW423825	F: CCATGCCAGATACAGTCGGTAGC<br>R: CAAGAACAGGGATTCTTGTTGACTC
PsPDH1	MW183670	F: TTGGGGTTGGGAAAGTGAATCAC<br>R: GTGCATCTGACATTCCACATAGTTG
PsProT1	MW030634	F: AGCAGCCAGTTGTCAAGAACATGATG<br>R: GCAATGACTGATTGAAGGAAGGCTG
PsProT2	MW030635	F: GGAAAGACAGCTAGAACAGAGAAG<br>R: CGAAGAGCTGAAATTGTGGTTGC
GAPDH	X73150	F: GTGGTCTCCACTGACTTTATTGGT<br>R: TTCCTGCCTTGGCATCAAA
β-Tubulin	X54844	F: GCTCCCAGCAGTACAGGACTCT<br>R: TGGCATCCCACATTTGTTGA
Actin	X90378	F: ATCATGGAGCCTGAGAGTTG<br>R: CACATACCACTAGGCCAATC

```
PsP5CS1         ------------------------------------------------------gacctg   6
MsP5CS1         ttttggagaaaacgtcatctcctttgggagttcttttaattattttcgagtctcgccctg   1414
MtP5CS1         ttttggagaaaacatcatctcctttgggagtccttttaattattttcgagtctcgccctg   1319
BnP5CS1         ttttggagaagacctcatccccattaggcgtactcctgattgtttttgagtctcgacctg   1342
AtP5CS1         tcttagagaagacctcatcaccattaggcgtacttctgattgtttttgaatcccgacctg   1629
                                                                      * ****

PsP5CS1         atgctcttgttcagatagcttccttggcaatacgaagtgggaatgggcttctcttgaaag   66
MsP5CS1         atgcattggtacagatagcttccttggcaatacgaagtgggaatgggcttctcttgaaag   1474
MtP5CS1         atgcattggtacagatagcttccttggcaatacgaagtgggaatgggcttctcttgaaag   1379
BnP5CS1         atgcacttgtacagatagcttcacttgctatccggagtggaaatggtcttctattgaagg   1402
AtP5CS1         atgcacttgtacagatagcttcacttgccatccgtagtggaaatggtcttctgctgaagg   1689
                ****  * ** ***********  * ** ** ** ***** ***** *****  **** *

PsP5CS1         gaggcaaggaggctaagagatcaaatgcaattttgcacaaagtaattactgaagctatac   126
MsP5CS1         gaggcaaggaggctaatcgatcaaatgccattttgcacaaagtaattactgaagccatac   1534
MtP5CS1         gaggcaaggaggctaatcgatcaaatgccattttgcacaaagtaattactgaagccatac   1439
BnP5CS1         gtggaaaggaggccaggagatcaaatgctatcttacacaaggtgataactgatgctattc   1462
AtP5CS1         gtggaaaggaggcccggcgatcaaatgctatcttacacaaggtgatcactgatgcaattc   1749
                * ** ********     ********** ** ** ***** ** ** ***** ** ** *

PsP5CS1         cagatactgttggttcaaaacttattggacttgtgacatcaagagcagaaatacccgagc   186
MsP5CS1         ctgatactgttggttcaaaacttattggacttgtgacatcaagagctgaaatacctgagc   1594
MtP5CS1         ctgatactgttggttcaaaacttattggacttgtgacgtcaagagctgaaatacctgagc   1499
BnP5CS1         cagagactgttggtggtaaactcattggacttgtgacttcaagggaagagattcctgatt   1522
AtP5CS1         cagagactgttgggggtaaactcattggacttgtgacttcaagagaagagattcctgatt   1809
                * ** ********    ***** ************** ***** *  ** ** ** **

PsP5CS1         tacttaagttggatgatgtaattgatctggtgattccaagaggcagcaacaaacttgttt   246
MsP5CS1         tacttaagctggatgatgtaattgatctggtgattccaagaggcagcaataaacttgttt   1654
MtP5CS1         tacttaagctggatgatgtaattgatctggtgattccaagaggcagcaataaacttgttt   1559
BnP5CS1         tgctcaagcttgatgacgttattgatcttgtgatcccaagaggcagcaacaagcttgttt   1582
AtP5CS1         tgcttaagcttgatgacgttatcgatcttgtgatcccaagaggaagcaacaagcttgtta   1869
                * ** *** * ***** ** ** ***** ***** ******** ***** ** ******

PsP5CS1         ctgatatcaaaagttccacgaaaatccctgttctgggtcatgctgatggaatttgtcacg   306
MsP5CS1         ctgatattaagagttcgacgaaaatccctgttctaggtcatgctgatgggatttgtcacg   1714
MtP5CS1         ctgatattaagagttcgacgaaaatccctgttctaggtcatgctgatggaatttgtcacg   1619
BnP5CS1         cccagattaaaaacactacaaaaatccctgttcttggtcatgctgatggaatctgtcatg   1642
AtP5CS1         ctcagataaaaaatactacaaaaatccctgtgctaggtcatgctgatggaatctgtcatg   1929
                *  * ** ** *   * ** *********** ** ************** ** ***** *

PsP5CS1         tctatgttgataaatctgcaaatctggagatggctaagcagattgtactcgatgcaaaaa   366
MsP5CS1         tctatgttgataaatctgctaatttggagatggctaagcagattgtactcgatgcaaaaa   1774
MtP5CS1         tctatgttgataaatctgctaatttggagatggctaagcagattgtactcgatgctaaaa   1679
BnP5CS1         tatatgtggacaaggcttgtaatgtggatatggctaaacgcatagtttctgatgcaaagt   1702
AtP5CS1         tatatgtcgacaaggcttgtgatacggatatggcaaagcgcatagtttctgatgcaaagt   1989
                * ***** ** **  **    **  *** ***** ** *  ** **    ***** **

PsP5CS1         cagattatccagcaggctgcaatgccatggaaacacttcttgttcataaggatttggtag   426
MsP5CS1         cagattgtccatcaggatgcaatgccatggaaacacttcttgttcataaggatttggtag   1834
MtP5CS1         cagattatccatcaggatgcaatgccatggaaacacttcttgttcataaggatttggtag   1739
BnP5CS1         tagactatccagcagcttgtaatgccatggaaactcttcttgtgcataaggatctggagc   1762
AtP5CS1         tggactatccagcagcctgtaatgcgatggaaacccttcttgtgcataaggatctagagc   2049
                  ** * **** ***  ** ***** ******** ******** ********* * *

PsP5CS1         agaaaggctggataaataatattatcgtcgacttgcggacagaaggcgttacgatatatg   486
MsP5CS1         agaaaggctggctcaatagtattagtgacgatttgcggtcggaaggtgttactttatatg   1894
MtP5CS1         agaaaggctggctcaatagtattagtgtcaatttgcggtcagaaggtgttacgttatatg   1799
BnP5CS1         agaacgctgtgctcaatgagcttatatttgctctgcagagcaatggagtcactttgtatg   1822
AtP5CS1         agaatgctgtgcttaatgagcttatttttgctctgcagagcaatggagtcactttgtatg   2109
                **** *    * * ***    ***         *** *    * ** ** **  * ****

PsP5CS1         gaggacccaaagcaagttctctattagctattccacttgctcgttcactacatcacgagt   546
MsP5CS1         gaggaccgaaagcaagttctctgttgaatgttccacttgctcgttcattacatcatgagt   1954
MtP5CS1         gaggaccgaaagcaagttctctgttgaatgttccacttgctcgttcattacatcacgagt   1859
BnP5CS1         gtggaccaaaggcaagtaaaatacttaacctaccagaagcacggtcgttcaatcacgagt   1882
AtP5CS1         gtggaccaagggcaagtaagatactgaacataccagaagcacggtcattcaaccatgagt   2169
                * ***** *  ******    *  *     * ***   ** ** **  *  * ** ****
```

```
PsP5CS1      attgttcgttggcttgcactgttgaaatcgttgatgatgtttatgcagctatcggtcata   606
MsP5CS1      actgttcgttggcttgcaccttggaaatcgtggatgatgtttatgcagctattcatcata   2014
MtP5CS1      actgttcgttggcttgcaccgtggaaattgtggatgatgtttatgcagctattgatcata   1919
BnP5CS1      actgttccaaggcgtgcactgttgaagttgtagaagatgtttatggtgctgtagatcaca   1942
AtP5CS1      actgtgccaaggcttgcactgttgaagttgtagaagacgtttatggtgctatagatcaca   2229
             * *** *   *** *****  * *** * ** ** ** *******  *** *   *** *

PsP5CS1      taaatctttatggaagtgcacatactgattccattatcacggaagatcatgaagtagctg   666
MsP5CS1      taaatctttatggaagtgcacataccgattccattgtcacagaagatcatgaagtagctg   2074
MtP5CS1      taaatcgttatggaagtgcacataccgattccattgtcacagaagatcatgaagtagcta   1979
BnP5CS1      ttcaccgacatggaagtgcacacacagactgtattgtgacagaggatcccgaagttgcag   2002
AtP5CS1      ttcaccgacatgggagtgcacacacagactgcattgtgacagaggatcacgaagttgcag   2289
             *  * *   **** ******** ** ** *  *** * ** ** ****  ***** **

PsP5CS1      atgtatttcttcgtcaagtcgacagtgctgccgtatttcacaatg---------------   711
MsP5CS1      atgtatttcttcgtcaagtcgacagtgctgctgtttttcacaatgctagtacaagattca   2134
MtP5CS1      atgtatttcttcgtcaagtcgatagtgctgctgtttttcacaatgctagtacaagattca   2039
BnP5CS1      agctattccttcgccaagtggacagcgctgctgtgttccacaacgcaagcacaagattct   2062
AtP5CS1      agctattccttcgccaagtggatagcgctgctgtgttccacaacgccagcacaagattct   2349
             *  **** ***** ***** ** ** ***** ** ** ***** *
```

Figure S1. The alignment of the nucleotide sequence fragment of *Pisum sativum PsP5CS1* (MW030636) with the sequences of *P5CS1* genes from other plants. *Arabidopsis thaliana* (*AtP5CS1*, NM_129539), *Brassica napus* (*BnP5CS1*, AF314811), *Medicago sativa* (*MsP5CS1*, X98421), and *Medicago truncatula* (*MtP5CS1*, AJ278818). Identical nucleotides are marked by black asterisks.

```
BnP5CS2      aggacgaggtggtatgactgcaaaagttaaagctgctgttaatgcagcttatggtggcat   883
AtP5CS2      aggacgaggggggtatgactgcaaaagttaaagctgctgttaatgcagcttatggtggcgt   1067
PsP5CS2      ----------------actgctaaggttaatgctgctgttgctgctgctaatgctagtat   44
MtP5CS2      gggcagaggggggtatgactgctaaggttaatgctgctgtttgtgctgcttattcaggcac   830
                             ***** ** ***** *********  *** *** **     *

BnP5CS2      tcctgttatcataaccagtgggtatgcagctgagaatatagctaaagttcttaaaggact   943
AtP5CS2      tcctgttatcataaccagtggatatgcagctgagaatataagtaaagtccttagaggact   1127
PsP5CS2      ccctgttattataattagtggctatgccacagacaacatcatacgagtgcttcaaggga   104
MtP5CS2      acctgtgattatcaccagtggctatcctaatgacaacatcatgcgagtgcttcaaggaga   890
              ***** ** ** *  ***** *** *    ** ** **      *** ***  ***

BnP5CS2      gcgtgttggtaccttgttccaccaagatgcacatttgtgggctagagtcgtagatactac   1003
AtP5CS2      gcgtgttggtaccctgttccatcaagatgctcatttatgggctccggtcgtagatactac   1187
PsP5CS2      gaaaataggtaccgtctttcataaagatgcacatctgtggaagagcttaaaggaagagac   164
MtP5CS2      aagaattggtactgtattccataaggatgctcacttgtggaccagcataaaggaaataac   950
                  * *****  * ** **  * ***** **  * ***       *    **    **

BnP5CS2      ttctcgtgacatggcagttgctgcaagggaaagttctagaaagcttcaggctttgtcttc   1063
AtP5CS2      ttctcgtgacatggcagttgctgcaagggaaagctcaagaaagcttcaggccttatcttc   1247
PsP5CS2      tgcacatgaaatggcagttgcagcacgtaatagttctagacgtcttcaggccctaaaatc   224
MtP5CS2      tgcacatgaaatggcagttgcagcacgcgacagctctaggaagcttcagattctaaattc   1010
             * * * *** *********** *** *  * ** ** **    *****    *    **

BnP5CS2      agaagataggaaaaacattctactagatatagccaacgctctagaagcaaatgagaaaat   1123
AtP5CS2      agaagataggaaacaaattctacacgacattgccaatgcccttgaagtaaatgagaaaac   1307
PsP5CS2      tgaagaaaggagggaagtattgctggccgtggctgatgcattggagaaaaaccaaaagtt   284
MtP5CS2      tgaacaaaggaagaaaatattgttggatgtggctgctgctatagagaaaaatgaaagtga   1070
              *** * ****   *  *  *    *   * **    **  * **   ***  * *

BnP5CS2      aattaaggctgagaatgatttagatgttgctgcagcacaagaagctggatatgaagagtc   1183
AtP5CS2      aattaaagctgagaatgatttagatgttgctgcagcacaagaagctggatatgaagagtc   1367
PsP5CS2      gataaagttagagaatgaggctgatattgctgatgcgttggaagccggatatgacaagtc   344
MtP5CS2      gatcaggcttgagaatgcttctgatgttgctgatgcagaggaggctggatatgagagatc   1130
              ** *     *******     *** ******  **    ** ** ********    **

BnP5CS2      tttggtagctcgtttagttatgaagcctggaaagatctcaagccttgcagcttctattcg   1243
```

```
AtP5CS2      tttggtagctcgcttagttatgaagcctgggaagatctcaagccttgcagcttccgttcg   1427
PsP5CS2      tctgatatcacgtttaaccctgaagcctgagaagatctctagtcttgcaaagtctgtgcg   404
MtP5CS2      actgatatcgcgtttgacactgagacctgagaagattgctagtcttgtaaagtctgttcg   1190
               ** ** * ** **     ***  ****  *****  * ** **** *   **  * **

BnP5CS2      ccagctagctgaaatggaagacccaattggtcgtgttctaaagaaaactgaggttgcaga   1303
AtP5CS2      ccagctagccgaaatggaagatccaataggccgtgtattaaagaaaactcaggttgcaga   1487
PsP5CS2      cgtgctggcagatatggaagaaccaattggccaggttttgaagagaactgagctagctga   464
MtP5CS2      caagctggcagaaatggatgaacccattggtcagattttgaagagaactgagatagcaga   1250
             *  *** ** ** ***** ** ** ** ** *   *  * **** **** ** * ** **

BnP5CS2      tggtcttattttagagaaaacctcatcaccattaggtgttcttctgattgtttttgaatc   1363
AtP5CS2      tgatcttattttagagaagacctcatcaccaataggtgttcttctgattgtttttgaatc   1547
PsP5CS2      tgacctcgtcttggagaaaatatcatgttctcttggtgtcctcttgattatatttgagtc   524
MtP5CS2      taaactcgttctggagaaaatatcatgtcccttgggtgtacttctagttatattcgagtc   1310
             *   **  *  * ***** *  ****   *  * ***** **  *  ** * ** ** **

BnP5CS2      ccgtcctgatgcacttgttcagatagcttcacttgcaatcaggagtggaaatggtcttct   1423
AtP5CS2      ccggcctgatgcacttgttcagatagcttcgcttgcaatccggagtggaaatggtcttct   1607
PsP5CS2      acgacccgatgctcttgttcagatagctgcattggcaattcgaagtggaaatggcttatt   584
MtP5CS2      tcgacctgatgctcttgttcagatagctgcattggcaattcgaagtggaaatggtttatt   1370
              ** ** ***** *************** *  * *****  * ***********  *  *

BnP5CS2      gctgaagggtggaaaagaggctcgtcgatcaaatgctatcttacacaaggtgatcactga   1483
AtP5CS2      gctgaagggtggaaaggaggctcgtcgatcaaatgctatcttacacaaggtgatcactga   1667
PsP5CS2      acttaaaggaggaaaggaagcccgaagatcaaatgcgatcttacacaagatcattacttc   644
MtP5CS2      gctcaaaggaggaaaggaagccaaaagatctaatgcagtcttacacaagattattacttc   1430
              ** ** ** ***** ** **     **** *****  *********** * ** ***

BnP5CS2      tgcaattccgaagactgttggaggtaaactcataggacttgtgacttcgagagatgagat   1543
AtP5CS2      tgcaattccggagactgttggaggtaaactcataggacttgtgacctcaagagaggagat   1727
PsP5CS2      agccatgccagatacagtcggtagcaaacttatcgggcttgtcacttcaagagatgaaat   704
MtP5CS2      agctataccagatacagttggtggcaaacttattggccttgtgacttcaagggaagcaat   1490
              ** ** **  * ** ** **  * ***** ** ** ***** ** ** ** ** *  **

BnP5CS2      tcctgatttgctcaagcttgatgatgttattgatcttgtgatcccaagaggcagcaacaa   1603
AtP5CS2      tcctgatttgctcaagcttgatgacgttattgatcttgtgatcccaagaggcagcaacaa   1787
PsP5CS2      tccagatctactcaagcttgatgatgtgatagatcttgtggtccctagaggcagtaataa   764
MtP5CS2      tcctgatctactcaagcttgatgacgtgatagatcttgtggtccctcgaggcagtaataa   1550
             *** *** * ************** ** ** ********* ****  ******* ** **

BnP5CS2      gcttgtttctcagataaaaaaactcaacgaaaatcccagtgctagggcatgcggatggtat   1663
AtP5CS2      gcttgtttctcaaataaaaaactcgacgaaaatcccagtgctaggccatgctgatggaat   1847
PsP5CS2      gctggtttctcaaatcaaggagtcaacaagaatccctgttcttggtcatgctgatggaat   824
MtP5CS2      gcttgtttctcaaatcaaggattctacaaagattcctgttctcggtcatgctgatggaat   1610
             *** ******** ** **  * ** ** *  ** ** ** ** ** ***** ***** **

BnP5CS2      ctgtcatgtgtatgttgacaagtcttgtaaagtagacatggcaaaacgtgttgtttcaga   1723
AtP5CS2      ctgtcatgtatatgttgataagtctggtaaactggacatggcaaagcgcattgtttccga   1907
PsP5CS2      ttgtcatatctatgttgacaagtctgctaatattgatatggcaaagcatatagttaggga   884
MtP5CS2      atgtcatgtatacattgacaaagcagctaatatcaatgtggcaaagaagattgttaagga   1670
              ****** * **  **** **  *   ***  *  *  *******     * ***   **

BnP5CS2      tgcaaagttagactatccagcagcctgtaacgctatggaaaccccttcttgtacataagga   1783
AtP5CS2      tgcaaagttggactatccagcagcctgtaatgcgatggaaaccccttcttgtacataaaga   1967
PsP5CS2      tgcaaagactgattaccctgcagcttgcaatgcattggaaaccccttcttgttcacaaaga   944
MtP5CS2      tgcaaagactgattatcctgcagcctgcaatgcaatggaaaccccttcttgttcacaaaga   1730
             *******   ** ** ** ***** ** ** **   *************** ** ** **

BnP5CS2      tctagagcagaatggtgtgctaaacgagcttatatatgccctgcaagccaatggtgtcac   1843
AtP5CS2      tttggagcagaatggttttctcgatgatcttatttatgttctgcaaaccaaaggcgtcac   2027
PsP5CS2      t-----------------------------------------------------------   945
MtP5CS2      tctagcaggaaatggtggactgaatgaacttgttgctgaactccaaaaacaaggtgttca   1790
             *
```

Figure S2. The alignment of the nucleotide sequence fragment of *Pisum sativum PsP5CS2* (MW423825) with the sequences of *P5CS2* genes from other plants. *Arabidopsis thaliana* (*AtP5CS2*, NM_115419), *Brassica napus* (*BsP5CS2*, AF314812), *Medicago truncatula* (*MtP5CS2*, JN809240). Identical nucleotides are marked by black asterisks.

```
AtPDH1      agctcatggaaggattcaagaaatctgtaggaaatgccaagagtccaatgtaccattgtt    1181
NtPDH2      agctcacaaaagacttacgaaaatttgcgagaaatgcttggaacacgacgttcctttact    890
MsPDH2      tgctaacaagagattccttgagctttgtcagaaatgtgtgcaagccaatattccattatt    1511
MsPDH1      tgctaacaagagattccttgagctttgtcagaaatgtgtgcaagccaatattccattatt    926
PsPDH1      -------------------------------------------------------ttatt    5
MtPDH1      agcaaaccaaagattacaacaactttgtaaaaaatgtgttgaagcaaacatgccaatatt    963
                                                                *  *

AtPDH1      gattgatgcggaagacacaatcctccaacccgcgatcgattacatggcttattcatcggc    1241
NtPDH2      cattgatgccgaggatacaactattcaacctggaattgattacatggcttattctgcagc    950
MsPDH2      ggttgatgctgaacatacttcagttcaacctgctattgattactttacttactcttctgc    1571
MsPDH1      ggttgatgctgaacatacttcagttcaacctgctattgattactttacttactcttctgc    986
PsPDH1      ggttgatgctgaacacactactgttcaaccggctatcgattacttcacgtattcttctgc    65
MtPDH1      ggttgatgctgaacacactactgttcaaccagctattgattacttcacatactcttcagc    1023
              ******* **  * **     * ***** *  ** ****** *  * ** **  * **

AtPDH1      gatcatgttcaatgctgacaaagaccgaccaatcgtttacaacacgattcaggcgtactt    1301
NtPDH2      aattaagtaccacaaagacg---atggccctttgattttcggaacaattcaagcttactt    1007
MsPDH2      tattatgcataacaaaggtg---aaaaccctattgtgtttggaaccattcagacttattt    1628
MsPDH1      tattatgcataacaaaggtg---aaaaccctattgtgtttggaacccttcagacttattt    1043
PsPDH1      gattgttcataacaaagatg---ataatcctattgtgtttggaacaattcaaacttactt    122
MtPDH1      aattatgcataataaagatg---ataatcctattgtgtttggaacaattcaaacttattt    1080
             **        *    *      *    **  *  * *     **  ****  * ** **

AtPDH1      gagagacgccggtgagagactgcatttggcagtacaaaatgctgagaaagagaatgttcc    1361
NtPDH2      gaaagacgcgaaagaaagaatggcgatagcgaaaaaagctgcagagaaaatgggagttcc    1067
MsPDH2      gaaagatgctaaggagagaatgttgttggcatcaaaggctgctgagaaaatggggatacc    1688
MsPDH1      gaaagatgctaaggagagaatgttgttggcatcaaaggctgctgagaaaatggggatacc    1103
PsPDH1      gaaagattctaaagagagattgtttcttgcgacacaagctgctgataaaagtggtattcc    182
MtPDH1      gaaagatgctaaggagagattgtttcttgcaacaaaagctgctgaaaaaattggaattcc    1140
            ** ***  *    ** *** **    * **   * *   *** ** ***       * **

AtPDH1      tatggggttcaagttggtgagaggggcttacatgtctagcgaagctagcttggcggattc    1421
NtPDH2      aatgggatttaagttggtgaggggtgcttatatgtgtagtgagagagagttggcttctcg    1127
MsPDH2      aatggggtttaagttggttagaggtgcttatatgtctagtgaaagaaaattggctgctga    1748
MsPDH1      aatgggatttaagttggttagaggtgcttatatgtctagtgaaagaaaattggctgctga    1163
PsPDH1      aatgggatttaagttggttagaggtgcttatatgtctatggaaagtaaaatagctgaatc    242
MtPDH1      tatgggatttaaattggttagaggtgcttatatgtcaacagaaagtacattggctgaatc    1200
             ***** ** ** ***** ** ** ***** ****  *  **        * **

AtPDH1      cctgggttgcaagtcgccagtc-cacgacacaattcaggatactcactcttgttacaatg    1480
NtPDH2      attaggattccaatctccaatt-catgatagcattgaacaaacacatgcttgcttcaatt    1186
MsPDH2      tttgggttatgcttctccaattacataacactattagggatactcataagtgtttcaatg    1808
MsPDH1      tttgggttatgcttctccaatt-cataacactattaaggatacacataagtgtttcaatg    1222
PsPDH1      tttcggatatggatcgccgatt-catgatactattcaagatacacatagtaacttcaatg    301
MtPDH1      ttttgggtctaagtcaccaatt-catgatactattaaagatacacataattgcttcaatg    1259
              * ** *     ** **  *  **  * *  ***    * ** **       * ****

AtPDH1      attgtatgacattcctgatggagaaagcatcaaacggttctggtttcggtgtcgttctcg    1540
NtPDH2      cttgcgctgagtttatgattgaagagattgctaatggctctggagcggttgtt---cttg    1243
MsPDH2      attgttcaaattatatgcttgagaagattgctaatggtcctggtggagttgtt---cttg    1865
MsPDH1      attgttcaaattacatgcttgagaagattgctaatggtcctggtggagttgtt---cttg    1279
PsPDH1      attgttcgtctttttttgcttgagaaggttgcaaatggacgtggttctgtcgtc---cttg    358
MtPDH1      attgttcatcttatttgcttgagaagtttgctaatggaaaggatctgttgtt---cttg    1316
             ***        *   ** * **  *     * ** **    **    *  **    ** *

AtPDH1      caacacataacgctgattc-----------------------------------------    1559
NtPDH2      ctactcataacattgagtcaggaaaacttgctgca-------------------------    1278
MsPDH2      caactcataacattgaatcaggtatatattatatgatatgatataatgatttatatttat    1925
MsPDH1      caactcataacattgaatc-----------------------------------------    1298
PsPDH1      ctacacataatattgaatc-----------------------------------------    377
MtPDH1      ctacacacaacattgaatc-----------------------------------------    1335
            * ** ** **   *** **
```

Figure S3. The alignment of the nucleotide sequence fragment of *PsPDH1* (MW183670) with the sequences of stress-downregulated *PDH* genes from other plants. *Arabidopsis thaliana* (*AtPDH1*, NM_001339059), *Medicago sativa* (*MsPDH1*, AY556386; *MsPDH2*, AY615900), *Medicago truncatula* (*MtPDH1*; XM_013595747), and *Nicotiana tabacum* (*NtPDH2*, AY639146). The identical nucleotides are marked by black asterisks.

```
GmProT1     cacattaggtatagagatcttgctggatttatatacggtaagaaagcgtataatctcaca   511
PsProT1     ---------------------------------------aaaaaggcatataaactcaca   21
MtProT1     cacattagatacagagatcttgctggatatatatacggcaaaaaggcatattctatcact   547
AtProT2     cacattcgttacagagatcttgcaggcttcatctatggtaaaaagatgtatcgtgttaca   490
AtProT1     catattcgctatagagaccttgctggattcatttacggtaggaaggcttatcatcttaca   387
AtProT3     cacattcgctatagagaccttgcgggattcatttacggtagaaaggcttattgtcttaca   836
                                                   *  **    ***    * **

GmProT1     tgggttctgcagtatatcaatcttttcatgataaatactggctacatcattttggccggt   571
PsProT1     tgggctcttcagtatatcaatctttttatgataaatactggcttcattattctggctggt   81
MtProT1     tggactctgcagtatatcaatcttttcatgataaatactggttacatcattttagctggt   607
AtProT2     tggggattgcaatatgtcaatcttttcatgattaattgtggcttcatcatacttgctggt   550
AtProT1     tggggattgcaatatgttaatcttttcatgattaattgtggattcattatcctagctggt   447
AtProT3     tgggtgttgcaatatgtcaatcttttcatgattaattgtggtttcatcatactagctggt   896
            ***    * ** *** * ******** ***** ***  *** * *** **  * ** ***

GmProT1     tcagccttgaaagctacttatgttctattcagggatgatgggctgctgaaacttccatat   631
PsProT1     tcagccttaaaagctacttataccttgtttagggatgatgctcagctgaagcttccatat   141
MtProT1     tcagccttgaaggctgcttataccgtgtttcgagatgatggtgtactgaagcttccgtat   667
AtProT2     tcggccttaaaggctgtttatgtactttttagagatgatagtctcatgaaactgcctcac   610
AtProT1     tccgccttaaaggctgtttatgtgcttttcagggatgatcataccatgaaactacctcat   507
AtProT3     tccgccttaaaggctgtttatgtgctttttagggatgatcatgccatgaagttacctcat   956
            ** ***** ** ***  ****    * **  * ******       ****  * **  *

GmProT1     tgtattgccataggtggatttgtgtgtgcaatgtttgccatctgcattcctcatctttca   691
PsProT1     tgtatagttatagctggttttgtgtgtgcaatgtttgccatttgtattcctcatctttca   201
MtProT1     tgtattgctatagctggaattgtgtgtgcaatgtttgccatttgcattcctcatctttca   727
AtProT2     ttcatcgccatcgcgggtgttgtatgcgcgattttcgcaatcggtattcctcatttatca   670
AtProT1     tttatagccattgctggtttgatttgtgcgattttcgctattgggattcctcatttatcg   567
AtProT3     tttatagctattgcgggtctgatttgtgcggtttttgctattggaattcctcatttgtcg   1016
            *  ** *  ** *  **  *  * ** **  * ** ** **  * ********* * **

GmProT1     gctctgggaatttggctaggattttcaacagtcttcagcctggcatatattgttatatca   751
PsProT1     gctcttggaatttggttggcagtttcaaccgtcttcggcttggtatacatttccgtagca   261
MtProT1     gctcttggagtttggttgggagtttcaacagtcttcacctttgtttacattgtcatagca   787
AtProT2     gctcttggaatctggctaggagtttcaacaatcctcagcataatctacattattgttgca   730
AtProT1     gctcttggggtctggcttggagtttcgaccttcctcagtctcatctatattgttgtggca   627
AtProT3     gctcttgggatatggcttgcggtttcgactatcctcagcctcatctatatcgttgtggca   1076
            ***** **  * *** * *   **** **  ** **    *    ** **     *  **

GmProT1     tttgtgttgtcgcttaaagatggattacaatcaccgcctcgagattacgagattccagga   811
PsProT1     tctgttctatcaattaaagatggaatacattcaccgcctcgagattacaccattccagga   321
MtProT1     ttggtgctatcaattaaagatggaatgaattcacctgctcgagattacgctgttccagaa   847
AtProT2     atagttctatcagctaaagatggagtaaacaagcctgaaagagattacaacatacaagga   790
AtProT1     atcgtgctatcggttagagatggagtaaaaacaccttcaagagattacgagatacaagga   687
AtProT3     atcgtgctatcggttaaagatggagttaaagcaccttcaagagattacgagatacaagga   1136
               **  * **   ** ******* *  *    **     ********    * * ** *

GmProT1     gatggtgtaagcaaaatctttacaataattggagcatctgctaaccttgtgtttgcattt   871
PsProT1     gaaggggcaagtaaagtatttgaaacgattggggcgtctgctaacctagtgttcgcatat   381
MtProT1     catggtgtaactaaaatattcacaacaattggggcgtctgctagtctagtgttcgcatac   907
AtProT2     tcatcaataaacaaactctttaccataacaggagcagctgcaaatctagttttcgcattc   850
AtProT1     tcatcattgagcaaactttttaccatcacaggagcagccgcaaatctggttttcgcattc   747
AtProT3     tcaccactaagcaaactttttaccatcactggagcagccgctacactagttttcgtattc   1196
                     *  *** * **    *  *  ** **  * ** *  ** ** ** * **

GmProT1     aataccggcatgcttcctgagatacaggcaacaattaggcaaccagttgttaagaacatg   931
PsProT1     aacaccggcatgcttcccgagattcaggcaacaattaagcagccagttgtcaagaacatg   441
MtProT1     aataccggcatgcttcctgagatacaggccacaattaagcaaccagttgtcaagaacatg   967
AtProT2     aacacgggaatgctcccggaaatacaggccacggtgaagcaaccggtcgttaaaaacatg   910
AtProT1     aacaccgggatgcttccagaaattcaggcaacagtgaggcaaccagttgttaaaaacatg   807
AtProT3     aacacagggatgcttccagaaattcaggcaacagtaaagcaaccagtagtaaaaaaacatg   1256
            ** ** ** ***** ** ** ** ***** **  * * *** ** ** ** ** ******

GmProT1     atgaaagccttgtactttcagtttaccgttggagttctaccattgtacctggttgccttt   991
PsProT1     atgaaatccctttattttcagtttacagtcggagttataccattgtaccttataaccttt   501
MtProT1     atgaaatccctgtggtttcagtttaccattggacttgtaccaatgtacatggttaccttt   1027
AtProT2     atgaaggctctgtattttcaattcactgttggtgttttacctatgtacgcggttacattc   970
AtProT1     atgaaggctctatactttcaattcacagcgggtgttttaccgatgtacgcagttacattc   867
AtProT3     atgaaggctctatactttcaattcacggtgggtgttttaccgatgtttgcggttgtattc   1316
            *****  *  * *  ***** ** **    **  ** ****  ***      *    **
```

```
GmProT1         acaggatactgggcttatggatcttccacagaagtgtatttgctgaatagtgtgaatggt   1051
PsProT1         tcaggatattgggcttatggatcttccacagaaacctatttactgaatagtgtcaacggt   561
MtProT1         gcaggatactgggcttatggaaataagacagaaacctatttgctgaatagtgtgaacggt   1087
AtProT2         atcggatattgggcttacgggtcctcgacatcgacttatctcttaaacagcgtcagtgga   1030
AtProT1         atcggatattgggcttacggatcctcaacctcgacctatctactaaacagtgttaatggc   927
AtProT3         atcgggtattgggcttacggatcctcgacctcgccctatctactaaacaacgttaatggt   1376
                   ** ** ******** **       **       *** *  * ** *  ** *  **

GmProT1         ccagtttgggtgaaggcttctgccaacatcacagcctttcttcaatcagtcattgcattg   1111
PsProT1         ccggtctgggtgaaggctttcgccaatatttcagccttccttcaatcagtcattgc----   617
MtProT1         ccagcttgggtgaaagctttggccaatattacagccttccttcaatcagtcattgcatta   1147
AtProT2         cctgtttgggtcaaagcactcgctaacatttcagcttttctccaatctgttatctcttta   1090
AtProT1         ccactctgggtcaaagcccttgctaacgtctctgctatccttcaatctgttatctctttg   987
AtProT3         ccactttgggttaaagcccttgctaacatctccgctatccttcaatccgttatctcgttg   1436
                **    ***** ** **    ** **  *  * **  * ** ***** ** **  *
```

Figure S4. The alignment of the nucleotide sequence fragment of *PsProT2* (MW030635) with the sequences of *ProT* genes from other plants. *Arabidopsis thaliana* (*AtProT1*, X95737; *AtProT2*, X95738; *AtProT3*, NM_129215), *Glycine max* (*GmProT1*, XM_003552545), and *Medicago truncatula* (*MtProT1*, XM_003600790). Identical nucleotides are marked by black asterisks.

```
GmProT2         gaggaaggccctgtatttacaatatacagtgggagttctgttctattatggtgttaccgt   931
PsProT2         --------------------------------------------------------Ctat   4
MtProT2         gaggaaagccttgtactcacaatatacagtaggagccttgttttattatggtgttactat   1117
AtProT2         gatgaaggctctgtattttcaattcactgttggtgttttacctatgtacgcggttacatt   969
AtProT1         gatgaaggctctatactttcaattcacagcgggtgttttaccgatgtacgcagttacatt   866
AtProT3         gatgaaggctctatactttcaattcacggtgggtgttttaccgatgtttgcggttgtatt   1315
                                                                           *


GmProT2         aatgggatattgggcttatggatcaatggtgtccgcataccttcctgaaaacttaagtgg   991
PsProT2         tgttggatattgggcttatggatcaatggtatcttcctatcttcctgaaaatttaagtgg   64
MtProT2         tgttggatattgggcttatgggtcaatggtatcttcatatcttcctgaaaatctaagtgg   1177
AtProT2         catcggatattgggcttacgggtcctcgacatcgacttatctcttaaacagcgtcagtgg   1029
AtProT1         catcggatattgggcttacggatcctcaacctcgacctatctactaaacagtgttaatgg   926
AtProT3         catcgggtattgggcttacggatcctcgacctcgccctatctactaaacaacgttaatgg   1375
                  * ** *********** ** **       **  * ** **     * *   * * ***

GmProT2         tcccaaatggattgacgtcctcattaatgccatcgtctttttgcagtccatagtcaccca   1051
PsProT2         accaagatggatcaatgtcattgttaatgtcattgtttttctacaatccatagtttctca   124
MtProT2         tcctagatggatcaatgtgctcgttaatgtcatcgtctttcttcagtctgcagtttctca   1237
AtProT2         acctgtttgggtcaaagcactcgctaacatttcagcttttctccaatctgttatctcttt   1089
AtProT1         cccactctgggtcaaagcccttgctaacgtctctgctatccttcaatctgttatctcttt   986
AtProT3         tccactttgggttaaagcccttgctaacatctccgctatccttcaatccgttatctcgtt   1435
                 **    *** *  * *   *   ***       *   *  * ** **     *  *

GmProT2         acatatgtttgtagcaccaattcatgaggcattggacactaagttcctagaaattgacaa   1111
PsProT2         acatatgtttgtggcaccaattcacgaagctatggacacgagatttctcgaaatcggcaa   184
MtProT2         acatttatttgtggtaccaattcatgaggctttggacactagatttctagaaattggcaa   1297
AtProT2         acatatttttgcaagtccgacttatgagtatatggacacaaagtatggagtcaaaggaag   1149
AtProT1         gcacatatttgcgagtccaacatatgagtacatggacacaaagtatgggatcaaaggaaa   1046
AtProT3         gcacattttttgcaagtccaacatatgagtacatggacaccaagtttgggatcaaaggaaa   1495
                 **  * ****     ** *   * **     ******* *  *        *  *  *

GmProT2         ggccatgcattcaggggagaacttgaaacgcttatttctcctacgtgcgcttttctttac   1171
PsProT2         aggtatgcattcaggggagaatttcaaacgtttgtttcttctacgagtcttcttctatac   244
MtProT2         gggtatgcattcaggggagaatttgaaacgcttatttctcctaaggatgtgcttctatac   1357
AtProT2         tccattggcaatgaagaatctg---ttgtttagaacagtagcaagaggaagctacattgc   1206
AtProT1         cccatttgcgataaagaacttg---ctgtttaggatcatggcgagaggcgggtacatagc   1103
AtProT3         cccattggcattaaagaacttg---ttgtttaggatcatggcaagaggcgggtacatagc   1552
                     *         * *                    *     *       * *    *
```

```
GmProT2         agggaacacattcgttgctgcagcatttccttttatgggtgactttgtaaactttcttgg   1231
PsProT2         aggaaacacattcatcgcggccgcatttccttttatgggtgactttgtaaacttgttagg   304
MtProT2         tgggaatacattcattgctgcagcatttccttttatgggtgactttgtaaacttgctagg   1417
AtProT2         ggtgagcactcttctctctgcgcttttaccgtttctcggagatttcatgagccttaccgg   1266
AtProT1         ggtaagcacgcttatctcagcgctgctgccgttcctcggtgacttcatgagcctaactgg   1163
AtProT3         agttagtacgcttctctcagcactcttgccgttccttggggacttcatgagcctcactgg   1612
                 *  *  **  *  *  * **     * ** **  * ** ** **  * * * *    **

GmProT2         ctcattttcactcgttcctctgaccttcatgttccccagcatggtcttcatcaaggttaa   1291
PsProT2         ctcattttcacttgttcccctaacattcatgttcccaagcatgatttttcttaaggtaaa   364
MtProT2         ctcattttcactagttcctcttaccttcatgttcccaagcatgatctttcttaagataaa   1477
AtProT2         agcgataagcacgttccctctcacattcatattagcgaatcacatgtatcttgttgctat   1326
AtProT1         tgcagtgagcacattccctctcacattcattctagccaaccatatgtactataaggcaaa   1223
AtProT3         tgcagtgagcacattccccttacattcattctagccaaccacatgtactataaggctaa   1672
                  *  *        * ** ** ** *****  *  * *      * *           *

GmProT2         gggaagaacagctagaatagagaagaaggcgtggcactggttcaacatcgtttttcttt   1351
PsProT2         gggaaagacagctagaacagagaagaaggcatggcattggatcaacattatattttcatt   424
MtProT2         gggaaaaactgctagaacagagaagaaggtgtggcattggatcaacattgtcgtttcatt   1537
AtProT2         gaatgatgagcttagtcttgtgcaaaagctatggcattggctcaatgtttgcttctttgg   1386
AtProT1         gaacaataagctgaatgctatgcaaaagctatggcattggcttaacgttgtcttctttag   1283
AtProT3         gaacaataagctcaatactttgcaaaagctatgccattggctcaacgttgtcttcttcag   1732
                *            *       * * ***   ** ** *** * **  *     * *

GmProT2         tctgctcacaatagcaaccacaatttcagcaattcggttgatagtcaacaacatccaaaa   1411
PsProT2         tctacttacaatagcaaccacaatttcagctcttcg------------------------   460
MtProT2         tctacttacaatagcaaccacaatttcagctcttcggttcataatcaacaacgttcaaaa   1597
AtProT2         attaatgtctcttgctgctgctattgctgctgttagactcatctctgttgactccaagaa   1446
AtProT1         tttgatgtctgttgctgcagccattgcagctgtcagactcatcgccgttgattccaaaaa   1343
AtProT3         tttgatgtctgttgctgccgccattgcagctcttaggctcatcgcccttgactccaaaaa   1792
                  *  *  *  * **  *  * *** * **  *  *
```

Figure S5. The alignment of the nucleotide sequence fragment of *PsProT2* (MW030635) with the sequences *ProT* genes from other plants. *Arabidopsis thaliana* (*AtProT1*, X95737; *AtProT2*, X95738; *AtProT3*, NM_129215), *Glycine max* (*GmProT2*, XM_014775460), and *Medicago truncatula* (*MtProT2*, XM_013602264). Identical nucleotides are marked by black asterisks.

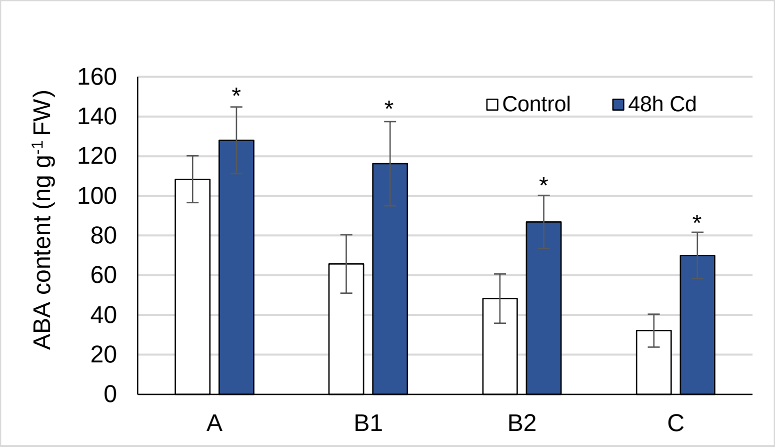

Figure S6. Changes in the abscisic acid (ABA) content after 48 hours of Cd treatment. Cadmium was applied as 50 µM $CdCl_2$. A, the youngest unexpanded leaves; B1, the youngest fully expanded leaves; B2, fully expanded mature leaves; C, the oldest true leaves. The results are the means (±SD) of three biological replicates. Asterisks indicate statistically significant differences between control and Cd-treatment ($P < 0.05$).

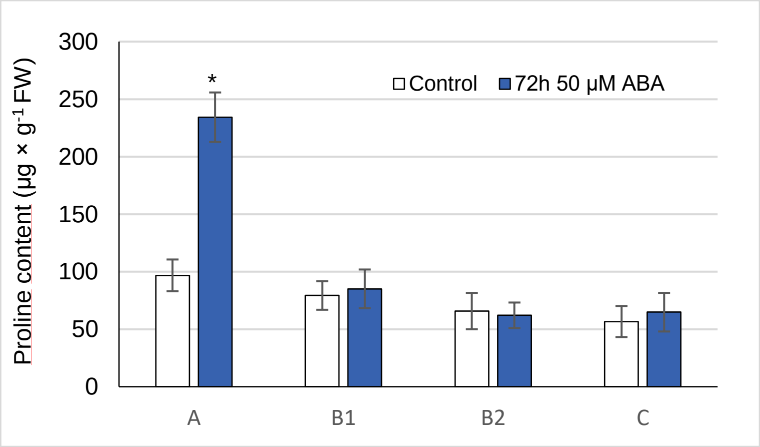

Figure S7. Changes in the proline content after 72 hours of abscisic acid (ABA) treatment. ABA was applied at concentrations of 50 µM. A, the youngest unexpanded leaves; B1, the youngest fully expanded leaves; B2, fully expanded mature leaves; C, the oldest true leaves. The results are the means (±SD) of three biological replicates. Asterisks indicate statistically significant differences between control and ABA-treatment ($P < 0.05$).

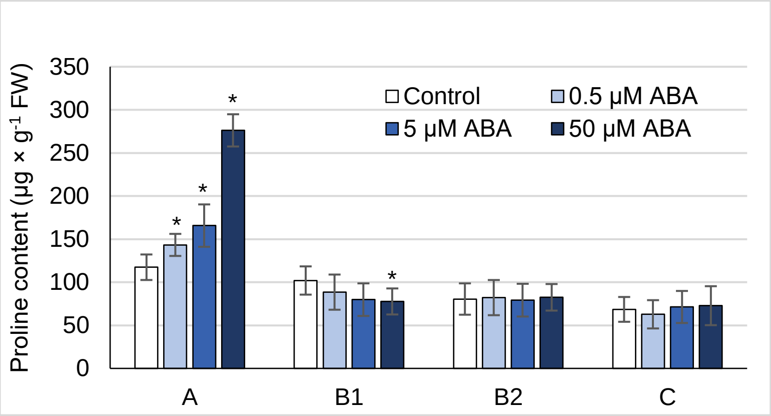

Figure S8. Changes in the proline content after 24 hours of abscisic acid (ABA) treatment. ABA was applied at concentrations of 0.5, 5, and 50 µM. A, the youngest unexpanded leaves; B1, the youngest fully expanded leaves; B2, fully expanded mature leaves; C, the oldest true leaves. The results are the means (±SD) of three biological replicates. Asterisks indicate statistically significant differences between control and ABA-treatment ($P < 0.05$).

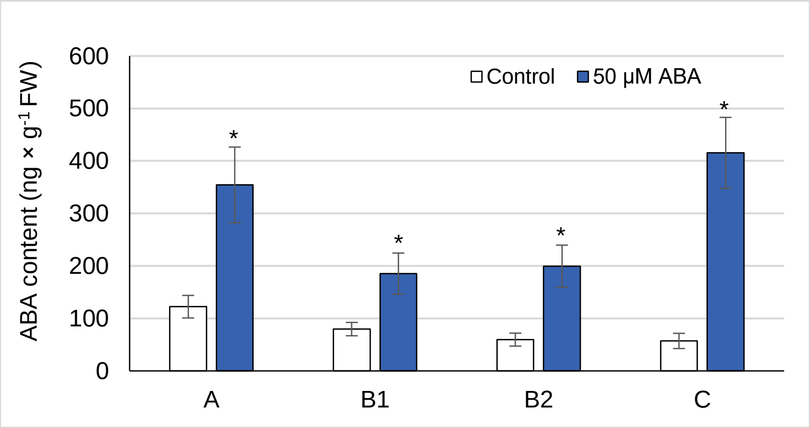

Figure S9. Changes in the abscisic acid content after 24 hours of abscisic acid (ABA) treatment. ABA was applied at concentrations of 0.5, 5, and 50 µM. A, the youngest unexpanded leaves; B1, the youngest fully expanded leaves; B2, fully expanded mature leaves; C, the oldest true leaves. The results are the means (±SD) of three biological replicates. Asterisks indicate statistically significant differences between control and ABA-treatment ($P < 0.05$).
